# Assessment of the Microbiological Acceptability of White Cheese (Akkawi) in Lebanon and the Antimicrobial Resistance Profiles of Associated *Escherichia coli*

**DOI:** 10.3390/antibiotics12030610

**Published:** 2023-03-19

**Authors:** Nasri Daher Hussein, Jouman W. Hassan, Marwan Osman, Khaled El-Omari, Samer A. Kharroubi, Imad Toufeili, Issmat I. Kassem

**Affiliations:** 1 Department of Nutrition and Food Sciences, Faculty of Agricultural and Food Sciences, American University of Beirut (AUB), Beirut 1107 2020, Lebanon; 2 Center for Food Safety, Department of Food Science and Technology, University of Georgia, 1109 Experiment Street, Griffin, GA 30223, USA; 3 Cornell Atkinson Center for Sustainability, Cornell University, Ithaca, NY 14853, USA; 4 Department of Public and Ecosystem Health, College of Veterinary Medicine, Cornell University, Ithaca, NY 14853, USA; 5 Quality Control Center Laboratories at the Chamber of Commerce, Industry & Agriculture of Tripoli & North Lebanon, Tripoli 1300, Lebanon; 6 Laboratoire Microbiologie Santé et Environnement (LMSE), Doctoral School of Sciences and Technology, Faculty of Public Health, Lebanese University, Tripoli 1300, Lebanon

**Keywords:** dairy, Akkawi, cheese, food safety, antimicrobial resistance, multidrug resistance, *Escherichia coli*, *Staphylococcus aureus*, Lebanon

## Abstract

Dairy foods are a staple in Lebanon, a low- and middle-income country that has been experiencing serious challenges to food safety and antimicrobial stewardship among other issues. The microbiological acceptability of dairy products has been of increasing concern. This is partially due to the failing economy and prolonged power outages that affect the quality of raw material and disrupt the dairy cold chain, respectively. Therefore, we assessed the microbiological acceptability of Akkawi, a popular white-brined cheese in Lebanon. For this purpose, we quantified the densities of *Escherichia coli* (a fecal indicator) and *Staphylococcus aureus* in cheeses collected from Lebanese retail stores. Additionally, we evaluated the antibiotic resistance profiles of the *E. coli* isolated from the cheese. *E. coli* and *S. aureus* were detected in 40 (80%) and 16 (32%) of the 50 cheese samples, respectively. Notably, 40 (80%) and 16 (32%) of the samples exceeded the maximum permissible limit of *E. coli* and *S. aureus*, respectively. A high percentage of the 118 *E. coli* isolated from the cheeses showed resistance to clinically and agriculturally important antibiotics, while 89 (75%) isolates were classified as multidrug-resistant (MDR). Given that Akkawi can be consumed without cooking, our findings highlight serious food safety and antimicrobial resistance problems that require immediate interventions.

## 1. Introduction

Dairy products are important components of a healthy and well-balanced diet [1]. White cheeses are particularly popular in many Mediterranean countries and are considered a nutritious staple food, providing proteins and micronutrients like calcium and phosphorous [2]. However, cheeses are high-risk foods that can be consumed without cooking, and contaminated cheeses can result in serious foodborne diseases. For example, several foodborne outbreaks were linked to cheese contaminated with *Escherichia coli*, *Salmonella* serovars, *Listeria monocytogenes*, and *Staphylococcus aureus* in Europe [3,4,5,6,7]. Additionally, several studies have reported the detection of *E. coli*, *S. aureus*, and *L. monocytogenes* in unpasteurized soft cheeses in the USA [2,8]. These contaminants pose a problem, because *S. aureus* is known to produce heat-stable enterotoxins that cause gastrointestinal disease [9], while *L. monocytogenes* is a major foodborne pathogen that is associated with severe health complications and deaths in pregnant women and immunocompromised individuals [10]. Similarly, pathogenic *E. coli*, such as Shiga toxin-producing *E. coli* O157:H7, can cause serious infections. Furthermore, it has been argued that foodborne pathogens are even more problematic in low- and middle-income countries that might suffer from under-developed public health systems [11].

The dairy market has a major agricultural and economic impact in Lebanon, being valued at $200 million USD and providing income for rural communities [12]. According to a report in 2016, 62,000 metric tons of dairy products are produced each year, with an average consumption of 24 kg of white cheese per capita in Lebanon [13]. Therefore, monitoring the safety of dairy products and ensuring the compliance of dairies are particularly important to stakeholders. However, Lebanon is currently facing major challenges to food safety due to weak governmental oversight, outdated national standards for microbiological and chemical contaminants, scarcity of baseline data, under-developed food safety monitoring systems, frail enforcement of food safety laws, and widespread pollution among others [14]. Due to these deficiencies, 42 outbreaks of foodborne disease, mainly associated with *Salmonella* infections, were reported in 2010, which is notable for a small country like Lebanon [15]. Additionally, the average number of reported food poisoning cases in Lebanon was 449 (±143) per year from 2012 to 2022 [16]. However, available data underestimate the reality of foodborne illnesses in Lebanon due to the lack of effective surveillance programs, and many foodborne pathogens, including *Shigella* spp., *Campylobacter* spp., *L. monocytogenes*, *Cryptosporidium* spp., norovirus, and hepatitis A virus among others, are not commonly investigated in clinical laboratories [17]. Furthermore, in the first ever nationwide study on food safety in Lebanon that was published in 2020, 28.7% of all tested foods were deemed microbiologically unsafe per the Lebanese ministry of public health [14]. The same report indicated that 28.3% of the dairy samples were microbiologically unacceptable, with bacterial pathogens such as *L. monocytogenes* being detected in cheeses. However, the prevalence of the pathogens and the contamination of different types of cheese were not thoroughly investigated. Given that Lebanon has been experiencing a protracted economic crisis, an influx of refugees (>1.5 million Syrian refugees), and shortages in medical supplies and hospital services [18,19,20], it is clear that foodborne illnesses will increase further, perpetuating the cycle of poverty and jeopardizing the well-being of the vulnerable, disenfranchised, and impoverished communities in Lebanon. Therefore, it is critical to assess the safety of important foods in Lebanon. However, only a limited number of studies have investigated the microbiological and chemical safety of cheese [14,21].

The problem of foodborne illness is aggravated by the rise of difficult-to-treat pathogens that acquire resistance to antimicrobial treatments [22]. Indeed, the extensive reliance on antibiotics in animal agriculture for the treatment and prevention of disease as well as for growth promotion has exacerbated the emergence of drug-resistant microorganisms [23]. The dissemination of multidrug-resistant (MDR) bacterial isolates in various food commodities, including milk and dairy products, is well documented in different countries [24,25,26,27]. Notably, the misuse of antimicrobials and the alarmingly high prevalence of drug resistance are also well-documented in the agricultural and environmental settings in Lebanon [28,29,30,31,32,33,34,35,36,37,38,39,40,41,42,43,44,45,46,47]. However, reports on the dissemination of antimicrobial resistance (AMR) in food commodities, especially dairy products, are scarce and these issues have not been thoroughly investigated in Lebanon [21,36]. Therefore, it was necessary to investigate the antimicrobial resistance profile of the bacterial contaminants in cheese in Lebanon.

The contamination of cheeses can occur during any step of processing, from milk production to cheese handling and storage. The densities of bacteria like *E. coli* and *S. aureus* are used as microbiological indicators to determine the acceptability and safety of cheese [9]. High bacterial densities of fecal indicators, like *E. coli,* also suggest the possible contamination of the food with enteric pathogens [48,49]. Additionally, the antibiotic resistance profiles of *E. coli* have been used as indicators for the emergence and spread of AMR in foods [50]. Taken together, we aimed to evaluate the microbiological safety of Akkawi, a highly popular white cheese, which we collected from the retail markets in Lebanon. Specifically, we quantified the prevalence and densities of *E. coli* and *S. aureus* in Akkawi cheese and compared them to the official cheese standards adopted by LIBNOR (Lebanese Standards Institute) [51,52]. Additionally, we assessed the antibiotic resistance phenotypes of the *E. coli* isolated from the cheese in order to evaluate the dissemination of AMR in this food product.

## 2. Results

### 2.1. Prevalence and Densities of E. coli and S. aureus

**Figure 1:**
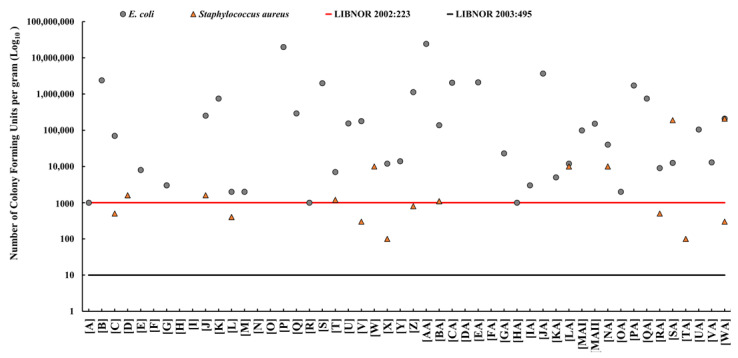
The prevalence and densities (colony-forming units per gram; CFU/g) of *Escherichia coli* (grey dot) and *Staphylococcus aureus* (orange triangle) in Akkawi cheese in Lebanon. The red line indicates the maximum allowable limit for *E. coli* and *S. aureus* (1000 CFU/g) according to the Lebanese Standards Institution (LIBNOR, standard 2002:223). The black line indicates the permissible limit of *E. coli* and *S. aureus* (10 CFU/g) according to the LIBNOR standard 2003:495.

*E. coli* was detected in 40 (80%) of the 50 cheese samples. The average densities of *E. coli* ranged between 1 × 10^3^ and 2 × 10^7^ CFU/g. The highest average density of *E. coli* was in Akkawi samples [AA] at 2 × 10^7^ CFU/g (Figure 1). *S. aureus* was detected in 16 (32%) of the cheese samples, with densities ranging between 1 × 10^2^ and 2 × 10^5^ CFU/g. The highest *S. aureus* density (2 x10^5^ CFU/g) was detected in sample [SA] (Figure 1). There was no statistically significant (*p* value > 0.05) correlation between *E. coli* and *S. aureus* densities. The identities of the *E. coli* and *S. aureus* isolates selected from positive cheese samples were further confirmed by PCR analysis.

### 2.2. Evaluation of Cheese Accessibility by Comparing E. coli and S. aureus Densities to LIBNOR Standards

According to LIBNOR 2002:223, the maximum allowable limit of *E. coli* and *S. aureus* in cheese is 1000 CFU/g; however, this limit was decreased to 10 CFU/g in the updated LIBNOR 2003:495. Subsequently, using *E. coli* densities, 40 (80%) of the cheese samples were found to be microbiologically unacceptable (unsafe) and would be rejected. Furthermore, 8 (16%) of the cheese samples exceeded the maximum allowable limit of 1000 CFU/g of *S. aureus* based on LIBNOR (2002:223). Using the updated LIBNOR (2003:495) standard, the number of rejected samples increased to 16 (32%) for *S. aureus* and remained at 80% for *E. coli (*Figure 1).

### 2.3. Antimicrobial Susceptibility Profiles of E. coli Isolated from Cheese Samples

**Figure 2:**
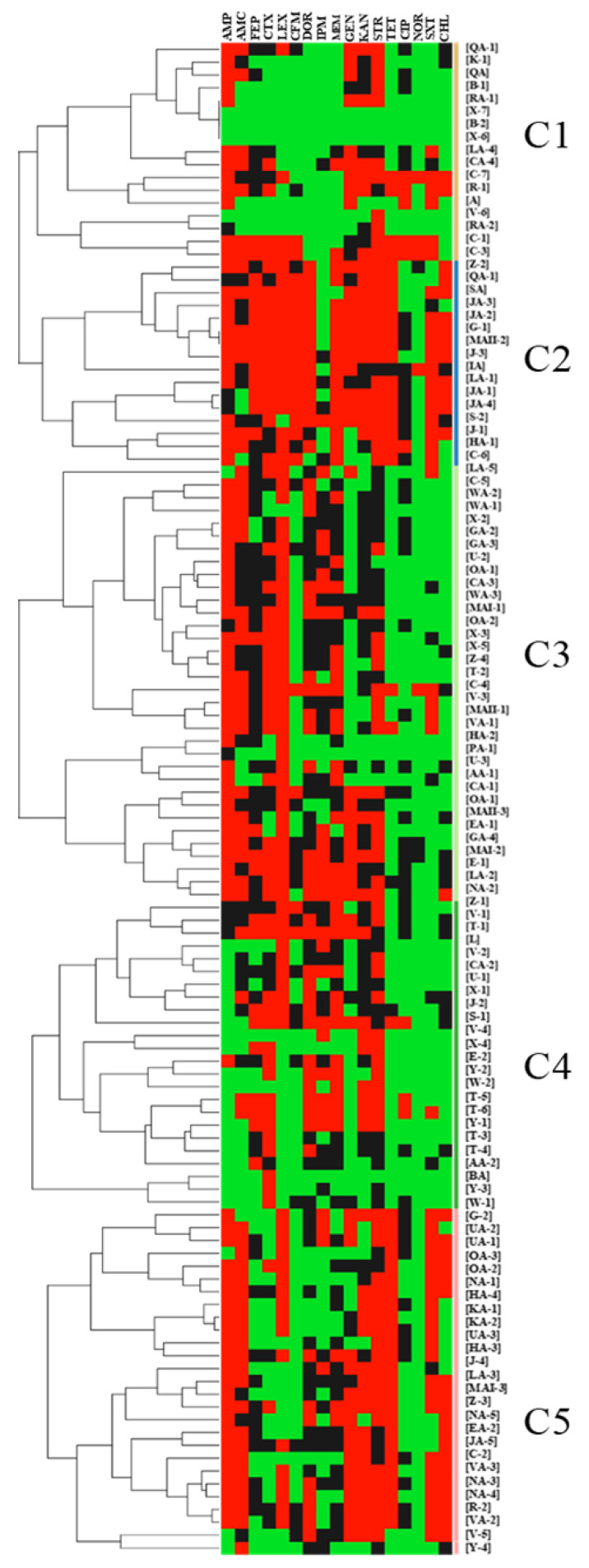
Hierarchical clustering of 118 *E. coli* isolated from Akkawi cheese in Lebanon. The isolate labels (IDs) refer to the associated cheese sample and the isolate number (if more than one *E. coli* was isolated from the same cheese sample). For example, [B-1] indicates that *E. coli* isolate #1 that was retrieved from Akkawi sample B. Squares in red represent resistance while those in black and green indicate intermediate resistance and susceptibility, respectively. The clusters are represented by the letter C.

**Figure 3:**
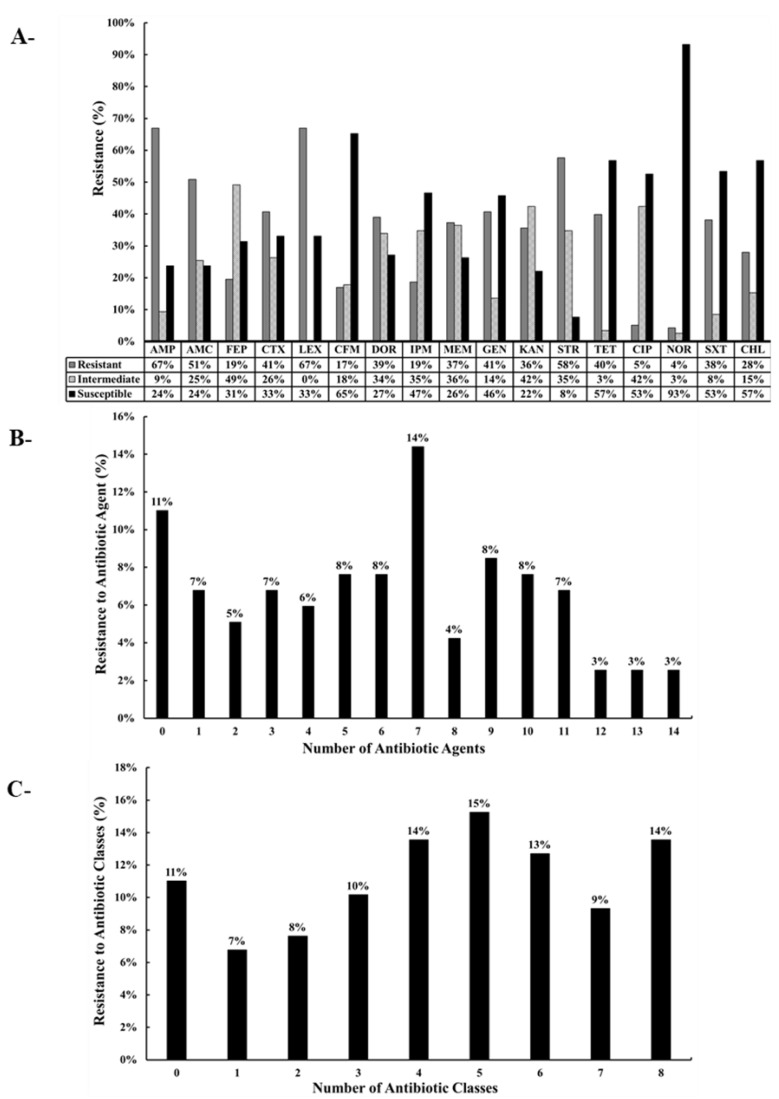
(**A**) Percentage of antibiotic-resistant of *E. coli* recovered from Akkawi cheese in Lebanon. Ampicillin (AMP), amoxicillin + clavulanic acid (AMC), cefepime (FEP), cefotaxime (CTX), cephalexin (LEX), cefixime (CFM), doripenem (DOR), imipenem (IPM), meropenem (MEM), gentamicin (GEN), kanamycin (KAN), streptomycin (STR), tetracycline (TET), ciprofloxacin (CIP), norfloxacin (NOR), trimethoprim + sulfamethoxazole (SXT), and chloramphenicol (CHL). The antibiotics are arranged according to the order of antibiotics/classes listed in the CLSI guidelines. (**B**) Percentage of isolates (%) resistant to the different antibiotic agents. (**C**) Percentage of isolates (%) resistant to different antibiotic classes.

The 118 *E. coli* isolates were resistant to the control antibiotics PEN and ERY as expected. Furthermore, among the 118 isolates, resistance was noted against AMP (67% of the isolates), AMC (51%), FEP (19%), CTX (41%), LEX (67%), CFM (17%), DOR (39%), IPM (19%), MEM (37%), GEN (41%), KAN (36%), STR (58%), TET (40%), CIP (5%), NOR (4%), SXT (38%), and CHL (28%) (Figure 2 and Figure 3). Intermediate resistance was observed against AMP (9%), AMC (25%), FEP (49%), CTX (26%), CFM (18%), DOR (34%), IPM (35%), MEM (36%), GEN (14%), KAN (42%), STR (35%), TET (3%,), CIP (42%), NOR (3%), SXT (8%), and CHL (15%) (Figure 2 and Figure 3). Notably, 89 of the 118 isolates (75%) were classified as MDR, thus showing resistance to at least three different classes of antibiotics. Furthermore, 16 (14%), 11 (9%), and 15 (13%) of the 118 isolates were resistant to 8, 7, and 6 classes of antibiotics, respectively (Figure 3C). Of particular concern was the observation that 67 of the 118 isolates (56.7%) were resistant to carbapenems (DOR, IPM, and/or MEM) (Figure 2 and Table 1). Five distinct clusters with different AMR profiles were identified by using the hierarchical cluster analysis (HCL) method, highlighting the diversity of the AMR phenotypes of the isolates. Resistance to AMP-AMC-GEN-KAN-STR was prominent in clusters 1 and 5. Most of the isolates that resisted 5 and more classes of antibiotics were placed in clusters 1, 2, and 5. Isolates that were resistant to carbapenems (DOR-MEM) were found in clusters 2 and 4, while resistance to aminoglycosides (GEN-KAN-STR) was mainly found in clusters 1, 2, 4, and 5. Notably, clusters 2 and 5 showed high resistance to trimethoprim-sulfamethoxazole and chloramphenicol (SXT-CHL) (Figure 2).

## 3. Discussion

Access to safe and nutritious food is necessary in order to maintain a healthy and productive community. Food safety is also a key to food security, which is especially critical in a country like Lebanon that has been experiencing increasing poverty due to an economic collapse. The latter has exacerbated pollution, the debilitation of infrastructure, and the proliferation of infectious diseases and antimicrobial resistance in the country [14,36,53]. Consequently, monitoring the safety of important foods is a critical need in Lebanon. Here, we focused on the safety of Akkawi, a white-brined cheese that is widely consumed (with or without cooking) in Lebanon and neighboring countries [54].

Our results revealed relatively high densities of *E. coli* and *S. aureus* in 80% and 32% of the cheese samples, respectively (Figure 1). We also found that 80% of the cheese samples would be rejected (deemed unacceptable for human consumption) based on the *E. coli* densities and LIBNOR standards (2002:223 and 2003:495), while up to 32% of the samples would be rejected based on *S. aureus* densities (Figure 1). Therefore, this study reported higher non-conformity rates in comparison to the limited studies available on dairy products in Lebanon [14,21]. Beyond the use of bacterial indicators, it is known that the enterotoxins secreted by *S. aureus* can cause gastrointestinal disease [9]. Given that a considerable number of people (~25% in some populations) carry *S. aureus* on their skin and/or in their nasal cavities, unhygienic practices while handling the cheese can lead to contamination [55,56]. This highlights the potential role of food handlers in the contamination of the cheese with important pathogens. Notably, it was estimated that *S. aureus* causes around 241,000 foodborne illnesses in the USA annually [55].

The high densities of *E. coli*, a fecal indicator, suggest the potential contamination of these cheeses with other enteric pathogens associated with fecal contamination [24,50]. Taken together, these results corroborated our previous analysis that showed that dairy foods, including cheeses, were at high risk of contamination with coliforms, *L. monocytogenes*, and *E. coli* [14]. Furthermore, fecal contamination can potentially occur at any stage of Akkawi cheese production. Although Akkawi is commonly produced from pasteurized milk, contamination can result from deficiencies in pasteurization as well as post-pasteurization processes that include hand-packing the cheese in draining hoops and curing it in brine solutions. Therefore, contamination can be attributed to the weak implementation of good hygienic practices among food handlers and food producers and/ or deficiencies in production and storage due to economic challenges that include unsustainable access to sources of electricity, clean water, and high-quality raw material.

Akkawi cheeses are not always sold in vacuum-sealed packages in Lebanon, especially those that are sourced from traditional and semi-modern dairies that usually provide the cheese in containers (buckets) filled with brine solution, and food handlers have to partition the cheese according to customers’ needs at the retail store. During sampling, we observed that the food handlers were wearing gloves, but that the gloves were used for extended periods of time and were not changed regularly. Moreover, the same cutting boards and knives were used to cut different cheese types and were not cleaned regularly, which increase the risk of cross-contamination. However, we found that even vacuum-sealed Akkawi cheese samples were contaminated with high densities of *E. coli* and *S. aureus*, which also suggested that the contamination might have occurred at the processing facilities prior to reaching the market, as explained earlier [25]. Beyond this, it is important to highlight that environmental pollution (e.g., contaminated water sources and pastures) can have major impacts on the safety and quality of dairy products. For example, a report in 2013 showed that the farming environment has a significant effect on the microbiological quality of milk [26], which would persist under deficient cheese production controls and processes. Taken together, strengthening food safety knowledge, endorsing the application of good personal hygiene, ensuring the availability of necessary factors (power, clean water and environment, and good quality raw material among others), establishing a sustainable contaminant-monitoring programs, and adherence to food safety standards are needed in order to maintain the safety of cheese and decrease the burden of foodborne illnesses.

The high prevalence of MDR *E. coli* in Akkawi cheese was not surprising (Figure 2 and Figure 3; Table 1), because several studies have reported a widespread antibiotic resistance in the Lebanese community, as well as in environmental and food matrices [29,34,35,37,38,39,46]. A recent study on dairy products in Lebanon screened 29 *E. coli* isolates that were found to be resistant to AMC (69% of isolates), CFM (11%), CHL (31%), and STR (100%), and all the tested isolates were multidrug-resistant [21]. However, in the aforementioned study, the number of *E. coli* isolates (n = 29) and antibiotics tested was very limited. In comparison, we tested 118 *E. coli* against 19 antibiotics that belonged to 9 different classes. In our study, the resistance against penicillins; AMP (67% of isolates), cephalosporins; FEP (19%,), CTX (41%), LEX (67%), CFM (17%), aminoglycosides; GEN (41%), KAN (36%), STR (58%), tetracyclines; TET (40%), and sulphonamides; SXT (38%) (Figure 3) was concerning but also expected, as these classes of antibiotics are heavily used in dairy cattle farming [57,58]. Our observations were further corroborated by a previous study that suggested that elevated levels of resistance against gentamicin can be attributed to the high use of this antibiotic in feed additives on Lebanese farms [59]. Additionally, another study conducted on dairy farms reported that aminoglycosides, which are normally used in clinical settings, are frequently used on animal farms in Lebanon [60]. Similarly, tetracycline was also reported to be extensively used in the Lebanese agricultural sector [60].

We noted that 56.7% of the *E. coli* isolates in our study were resistant to carbapenems, which is highly concerning. Carbapenems are considered last-resort antibiotics that are used as a salvage therapy to treat complicated MDR and extensively drug-resistant (XDR) Gram-negative infections [61]. When bacterial isolates develop resistance to carbapenems, colistin, one of the highest priority critically important antibiotics, is used as a viable treatment option [61]. However, the wide-emergence of resistance to colistin, due to the presence of the mobile colistin-resistant gene (*mcr),* has been well-document in Lebanon in various agricultural and environmental matrices [28,29,33,34,35,37,38,39,41,45,46]. The occurrence of resistance to critically important antibiotics in the food chain is a threat to public health and agriculture, heralding a rise in difficult-to-treat infections in both humans and farm animals. The high dissemination of antibiotic resistance in Lebanon has been attributed to the extensive reliance on and misuse of antibiotics in both animal farming and clinical settings [23]. It has been also established that there is a notable gap in knowledge about judicious antibiotic use among farmers in Lebanon [62]. For instance, dairy farmers believe that the use of antibiotics for prophylaxis and growth promotion is always beneficial, and that stopping the administration of antibiotics to their animals will lead to major economic losses [62]. Taken together, an immediate action plan is needed to address the dissemination of antibiotic resistance in Akkawi cheese and other food products.

## 4. Materials and Methods

### 4.1. Sample Collection

A total of 50 Akkawi cheese samples were aseptically collected from 16 major retail stores across Beirut, the capital of Lebanon, between September 2020 and October 2021. Forty-three cheese samples belonged to 9 brands, while 7 were unbranded. All samples were made from cows’ milk and collected from different lots. While Akkawi is normally made from pasteurized milk, this process was not evident in cases of unbranded cheese samples. The cheese samples were transported in a cooler with ice and processed immediately upon arrival at the laboratory (within 2 h of collection).

### 4.2. Sample Processing and Enumeration of E. coli and S. aureus

Each sample was aseptically processed by transferring 25 g of cheese to a sterile stomacher bag (Fisher Scientific, New Hampshire, USA) filled with 225 mL of buffered peptone water (BPW) (Oxoid, Hampshire, UK) [36]. The samples were then homogenized for 1 min at 250 rpms using a stomacher (Thomas Scientific, NJ, USA). The homogenates were 10-fold serially diluted (10^−1^, 10^−2^, 10^−3^, 10^−4^), and 100 µL of each dilution was plated in duplicate on the selective Chromogenic Tryptone Bile X-Glucuronic (TBX) agar (Oxoid, Hampshire, UK), and Baird-Parker (BPA) agar (Millipore-Sigma, Darmstadt, Germany) for the enumeration and isolation of *E. coli* and *S. aureus*, respectively. TBX plates were incubated at 37 °C for 24 h, and BPA plates were left for 48 h at 37 °C under aerobic conditions. After incubation, colonies that matched the diagnostic phenotypes of *E. coli* (Blue/Green colonies on TBX) and *S. aureus* (black shiny colonies with a halo on BPA) were counted, and the bacterial densities were reported as colony-forming units per gram of cheese (CFU/g) by averaging the counts from the duplicates.

The microbiological quality of the cheese (acceptability/rejection) was determined by comparing *E. coli* and *S. aureus* counts (CFU/g) to the two available standards published and adopted by LIBNOR (2002:223 and 2003:495). Specifically, standard (2002:223) indicates that the maximum permissible limit of *E. coli* and *S. aureus* in cheese is 1000 CFU/g [52]. Conversely, LIBNOR (2003:495) is more conservative, with a maximum allowable limit of 10 CFU/g [51]. *E. coli* and *S. aureus* isolates were purified, suspended in 1 mL Luria-Bertani (LB) broth (80%) (Oxoid, Hampshire, UK) with 0.5 mL glycerol (20%), and stored at −80 °C for further analysis [36].

### 4.3. Polymerase Chain Reaction (PCR) for the Confirmation of Identity of E. coli and S. aureus

Putative *E. coli* colonies were suspended in 100 µL of DNase-free water and placed in a water bath at 95 °C for 10 min to extract DNA [63]. The *E. coli* isolates were screened for a species-specific 16S rRNA gene fragment [64,65]. Briefly, PCR reactions (20 µL) were prepared, as reported elsewhere [38,39], and subjected to the following program: an initial denaturation at 95 °C followed by 38 cycles, each consisting of denaturation at 95 °C for 30 s, annealing at 58 °C for 45 s and extension at 72 °C for 1 min, followed by a final extension at 72 °C for 10 min [65]. The amplified PCR amplicons (544 bp) were visualized by gel electrophoresis. Reactions with DNase-free water instead of bacterial DNA were used as a negative control, while DNA extracted from *E. coli* DH5α was used as a positive control. Similarly, the PCR detection of a specific fragment of *femB* was used to confirm the identity of the *S. aureus* isolates [66]. However, the PCR program consisted of an initial denaturation at 95 °C for 4 min, followed by 30 cycles of denaturation at 94 °C for 1 min, annealing at 50 °C for 45 s, and extension 72 °C for 3 min, which was followed by a final extension at 72 °C for 10 min. *S. aureus* ATCC^®^ 6538 was used as a positive control.

### 4.4. Assessment of the Antibiotic Resistance Profiles of E. coli Using the Disk Diffusion Assay

The antibiotic resistance profiles of 118 *E. coli* isolates retrieved from the cheese (on average 3 *E. coli*, when available, were randomly selected from each positive cheese sample) were determined using the disk diffusion assay [67,68]. Pure *E. coli* colonies were suspended in Mueller–Hinton (MH) broth (Oxoid, Hampshire, UK). The optical density of the bacterial cultures was adjusted using the 0.5 McFarland standard and a spectrophotometer (Thermo Fisher Scientific, MA, USA). Thereafter, the cultures were spread on MH agar plates using swabs, as described in the guidelines of the Clinical and Laboratory Standards Institute (CLSI) [68]. Nineteen different antibiotic disks (Oxoid, Hampshire, UK) belonging to 9 different classes were then added to the MH agar plates. The antibiotics included penicillins: ampicillin (AMP; 10 μg); β-lactam/β-lactamase inhibitor combinations: amoxicillin/clavulanic acid (AMC; 20 μg/10 μg); cephalosporins: cefepime (FEP; 30 μg), cefotaxime (CTX; 30 μg), cefalexin (LEX; 30 μg), and cefixime (CFM; 5 μg); carbapenems: doripenem (DOR; 10 μg), meropenem (MEM; 10 μg), and imipenem (IPM; 10 μg); aminoglycosides: gentamicin (GEN; 10 μg), kanamycin (KAN; 30 μg), and streptomycin (STR; 10 μg); tetracyclines: tetracycline (TET; 30 μg); quinolones and fluoroquinolones: ciprofloxacin (CIP; 5 μg) and norfloxacin (NOR; 10 μg); folate-pathway inhibitors: trimethoprim/sulfamethoxazole (SXT; 25 μg); and phenicols: chloramphenicol (CHL; 30 μg) [36,59]. The MH agar plates were then incubated at 37 °C for 18 h. Penicillin (PEN; 6 µg) and erythromycin (ERY; 15 µg) were used as controls [36,53]. Additionally, *E. coli* DH5α and *E. coli* ATCC^®^ 25922 were used for quality control across the experiments. The diameters of the zones of inhibition around each antibiotic disk were measured and evaluated according to the CLSI and the European Committee on Antimicrobial Susceptibility Testing (EUCAST) standards [68,69]. The breakpoints that were used to determine the susceptibility of the isolates to the different antibiotics are listed in the supplementary Table S1. Additionally, the AMR profiles were analyzed using the hierarchical clustering (HCL) approach. Briefly, isolates were represented in rows and antibiotics in columns, while the AMR profiles were coded as follows: with 0 indicating resistance, 0.5 representing intermediate resistance, and 1 corresponding to susceptibility. Hierarchical clustering was then conducted using Morpheus (https://software.broadinstitute.org/morpheus/, accessed on 15 December 2022) by setting the Pearson correlation as a distance metric and choosing the complete linkage method [70]. A graphical HCL analysis was produced (heat map), with green colors representing susceptibility (1), while black and red colors corresponded to intermediate resistance (0.5) and resistance (0), respectively.

### 4.5. Statistical Analysis

A simple linear regression analysis was conducted using SPSS 23.0 (IBM^®^ SPSS^®^ Statistics, NY, USA) to evaluate the correlation between the bacterial densities in the Akkawi cheese samples. Statistical tests were two-sided, with a type I error set at α = 0.05.

## 5. Conclusions

This study highlights the frequent occurrence of a fecal indicator, *E. coli*, and *S. aureus* in Akkawi cheese collected from retail stores across Beirut, Lebanon. Given the popularity of Akkawi cheese among the Lebanese population, the high contamination with these bacterial indicators raises public health concerns and highlights the need for robust food safety systems. Subsequently, microbial pathogens such as *L. monocytogenes*, which have been previously associated with cheese samples [14], must be fully investigated in order to identify and mitigate the potential sources of contamination. Furthermore, the occurrence of MDR *E. coli* in the cheese samples emphasizes a paramount need to strengthen antimicrobial stewardship, revise agricultural practices, and adopt policies to curb the spread of antibiotic resistance. The latter is not only a local problem, because AMR can spread across national borders, causing a wide threat to multiple stakeholders. Consequently, efforts must focus on decreasing the over reliance on antibiotics in both agriculture and medicine to preserve the efficacy of these highly important dugs, while seeking viable and sustainable alternatives that do not promote the spread of resistance.

## Figures and Tables

**Table 1:** Antibiotic Resistance Profiles of 118 *E. coli* isolated from Akkawi cheese collected from Retail Stores in Beirut, Lebanon.

*E. coli* Isolate ID (n = 118) ^1^	Antibiotic Resistance Profile ^2^	Intermediate Resistance Profile
[B-2], [X-6], [X-7]	Pan-susceptible	No intermediate resistance
[A]	Pan-susceptible	AMP-GEN-CIP-SXT
[B-1]	AMP-STR	GEN-KAN-CIP
[C-1] *	AMP-AMC-FEP-CTX-LEX-CFM-STR-TET-CIP-NOR-SXT	GEN-KAN
[C-2] *	AMP-AMC-GEN-KAN-TET-SXT-CHL	STR-CIP
[C-3] *	AMP-AMC-FEP-CTX-LEX-CFM-MEM-KAN-STR-TET-CIP-NOR-SXT	GEN
[C-4] *	AMP-AMC-CTX-LEX-CFM-DOR-IPM-MEM-STR-TET-NOR-SXT	FEP-KAN-CHL
[C-5] *	AMP-AMC-LEX-DOR	FEP-CTX-CFM-MEM-STR-CIP
[C-6] *	AMP-CTX-LEX-CFM-MEM-STR-TET-SXT	FEP-IPM-CIP
[C-7] *	AMP-LEX-GEN-KAN-STR-TET-CIP-NOR-SXT-CHL	AMC-FEP-CTX
[E-1] *	AMP-FEP-CTX-LEX-DOR-IPM-MEM-GEN	AMC-CFM-KAN-STR-CIP-CHL
[E-2] *	AMP-CTX-DOR-MEM-STR	AMC-FEP-CFM-IPM-KAN
[G-1] *	AMP-AMC-FEP-CTX-LEX-CFM-DOR-MEM-GEN-KAN-STR-TET-SXT-TET	CIP
[G-2] *	AMP-LEX-IPM-GEN-KAN-STR-TET-SXT-CHL	DOR-CIP
[J-1] *	AMP-AMC-FEP-LEX-CFM-MEM-KAN-STR-TET-SXT-CHL	CTX-DOR-CIP
[J-2] *	FEP-CTX-LEX-DOR-MEM	AMC-CFM-IPM-KAN-STR-TET-CHL
[J-3] *	AMP-AMC-FEP-CTX-LEX-CFM-DOR-MEM-GEN-KAN-STR-TET-SXT-CHL	IPM
[J-4] *	AMP-AMC-IPM-GEN-KAN-STR-TET	DOR-MEM-SXT
[K]	AMP-GEN-STR	AMC-KAN-CHL
[L]	LEX-IPM	DOR-MEM-KAN-STR
[R-1] *	AMP-AMC-CTX-GEN-KAN-TET-CIP-SXT-CHL	FEP-CFM-STR
[R-2] *	AMP-AMC-LEX-DOR-GEN-KAN-STR- TET-SXT-CHL	FEP-CTX-CFM-MEM-CIP
[S-1] *	FEP-CTX-LEX-DOR-IPM-MEM-GEN-KAN-TET-CIP	CFM-STR-CHL
[S-2] *	AMP-CTX-CFM-DOR-IPM-MEM-GEN-KAN-STR-TET-SXT	AMC-FEP-CIP-CHL
[T-1] *	AMC-FEP-CTX-LEX-CFM-DOR-IPM-MEM-GEN-KAN	AMP-STR-CIP-CHL
[T-2] *	AMP-AMC-CTX-LEX-DOR-MEM	FEP-KAN-STR
[T-3]	CTX	FEP-DOR-MEM-KAN-STR
[T-4]	CTX-DOR	FEP-IPM-MEM-KAN-STR-CIP-CHL
[T-5]	Pan-susceptible	AMC-FEP-CTX-DOR-IPM-MEM-KAN-STR-CIP
[T-6]	Pan-susceptible	AMC-FEP-CTX-DOR-IPM-MEM-KAN-STR-CIP-SXT
[U-1]	LEX-DOR	AMC-CTX-MEM-KAN-STR
[U-2] *	AMP-LEX-MEM	AMC-FEP-CTX-DOR-IPM-KAN
[U-3] *	AMP-LEX-DOR-MEM	FEP-CTX-GEN-STR-CIP-CHL
[V-1] *	FEP-CTX-LEX-DOR-MEM-GEN-STR	AMP-AMC-CFM-IPM-KAN-CIP-CHL
[V-2]	LEX-STR	AMC-CTX-DOR-IPM-MEM-KAN
[V-3] *	AMP-AMC-CTX-LEX-STR-SXT	FEP-DOR-IPM-MEM
[V-4]	Pan-susceptible	IPM-KAN-STR
[V-5] *	DOR-GEN-KAN-STR-SXT-CHL	AMC-CFM-IPM-MEM
[V-6]	STR	No intermediate resistance
[W-1]	CTX	CFM-DOR-MEM-GEN-STR-CIP
[W-2]	Pan-susceptible	DOR-MEM-KAN-STR
[X-1] *	AMC-CTX-LEX-DOR-MEM-STR	FEP-IPM-GEN-KAN-SXT-CHL
[X-2] *	AMP-AMC-LEX	CTX-DOR-IPM-MEM-KAN-STR-CIP
[X-3] *	AMP-AMC-FEP-CTX-LEX-STR	DOR-IPM-MEM-KAN-SXT
[X-4]	Pan-susceptible	FEP-CTX-KAN-STR
[X-5] *	AMP-CTX-LEX-MEM-STR	AMC-FEP-DOR-IPM-KAN-CHL
[Y-1]	Pan-susceptible	FEP-CTX-DORIPM-MEM-KAN-STR
[Y-2]	Pan-susceptible	CTX-DOR-IPM-MEM-STR
[Y-3]	CTX	IPM-STR
[Y-4]	AMC	DOR-IPM-STR-CHL
[Z-1] *	LEX-CFM-MEM-STR	AMP-AMC-FEP-CTX-DOR-IPM-KAN-CIP
[Z-2] *	AMP-AMC-CTX-LEX-DOR-MEM-GEN-KAN-STR-TET-CHL	FEP-CFM-NOR
[Z-3] *	AMP-AMC-CTX-DOR-MEM-GEN-KAN-STR-TET-SXT-CHL	FEP-IPM
[Z-4] *	AMP-CTX-LEX-MEM-STR	AMC-FEP-DOR-IPM
[AA-1] *	AMP-CTX-LEX-MEM	DOR-IPM-SXT
[AA-2]	FEP	CTX-DOR-IPM-MEM-STR-SXT
[BA]	Pan-susceptible	CTX
[CA-1] *	AMP-AMC-LEX-CFM-GEN-KAN-STR	FEP-CTX-DOR-IPM-MEM-TET-CIP
[CA-2] *	LEX-DOR-IPM-MEM-STR	AMC-FEP-CTX-CFM-KAN
[CA-3] *	AMP-CTX-LEX-DOR-IPM-MEM	AMC-FEP-KAN-STR-SXT
[CA-4] *	AMP-AMC-CTX-MEM-GEN-KAN-STR	FEP-IPM-CIP-SXT
[EA-1] *	AMP-AMC-FEP-LEX-IPM-GEN-STR	DOR-KAN
[EA-2] *	AMP-AMC-GEN-KAN-TET-CHL	FEP-DOR-IPM-MEM-STR
[GA-1] *	AMP-LEX-IPM	AMC-FEP-CTX-DOR-MEM-KAN-STR
[GA-2] *	AMP-AMC-LEX-IPM	CTX-DOR-MEM-KAN-STR-CIP
[GA-3] *	AMP-CTX-LEX-IPM-STR	AMC-FEP-CFM-DOR-MEM-KAN-CIP
[GA-4] *	AMP-AMC-CTX-LEX-IPM-GEN-STR	FEP-CFM-DOR-MEM-KAN-CIP-NOR
[HA-1] *	AMP-AMC-LEX-DOR-MEM-STR-TET-CIP-SXT	FEP-CTX-CFM-KAN
[HA-2]	AMP-LEX	AMC-FEP-MEM
[HA-3] *	AMP-AMC-LEX-DOR-MEM-GEN-STR-TET-SXT	FEP-CTX-IPM-KAN-CIP
[HA-4] *	AMP-AMC-LEX-KAN-STR-TET-SXT	MEM-CIP
[IA] *	AMP-FEP-CTX-LEX-CFM-DOR-MEM-GEN-NOR-SXT	AMC-KAN-STR-TET-CIP-CHL
[JA-1] *	FEP-CTX-LEX-CFM-DOR-MEM-GEN-KAN-STR-TETSXT-CHL	AMP-CIP
[JA-2] *	AMP-FEP-CTX-LEX-CFM-DOR-MEM-GEN-KAN-STR-TET-SXT-CHL	AMC-CIP
[JA-3] *	AMP-FEP-CTX-LEX-CFM-DOR-MEM-GEN-KAN-STR-TET	AMC-SXT
[JA-4] *	FEP-CTX-LEX-CFM-DOR-MEM-GEN-KAN-STR-TET-SXT-CHL	AMP-IPM-CIP
[JA-5] *	AMP-AMC-LEX-GEN-KAN-TET-CHL	FEP-CTX-CFM-DOR-IPM-MEM-STR
[KA-1] *	AMP-AMC-LEX-KAN-STR-TET-SXT	GEN
[KA-2] *	AMP-AMC-LEX-KAN-STR-TET-SXT	GEN-CIP
[LA-1] *	AMP-FEP-CTX-LEX-CFM-DOR-MEM-STR-TET-SXT-CHL	AMC-IPM-GEN-KAN-CIP
[LA-2] *	AMP-AMC-CTX-LEX-DOR-IPM-MEM-GEN-STR	FEP-CFM-KAN-TET-CIP-CHL
[LA-3] *	AMP-AMC-KAN-STR-TET-SXT-CHL	FEP-DOR-IPM-MEM-GEN
[LA-4] *	AMP-AMC-GEN-SXT	FEP-CTX-MEM-KAN-STR-CIP
[LA-5] *	AMC-CTX-LEX-IPM-GEN-SXT	FEP-DOR-STR
[MAI-1] *	AMP-AMC-CTX-LEX-DOR-IPM-MEM-KAN-STR	FEP-GEN
[MAI-2] *	AMP-AMC-FEP-CTX-LEX-DOR-IPM-GEN-KAN-STR	CFM-MEM-CIP-NOR-CHL
[MAI-3] *	AMP-GEN-KAN-STR-TET-SXT-CHL	AMC-DOR-MEM
[MAII-3] *	AMP-AMC-CTX-LEX-DOR-MEM-STR-SXT	FEP-IPM-CIP
[MAII-2] *	MP-AMC-FEP-CTX-LEX-CFM-DOR-MEM-GEN-KAN-STR-TET-SXT-CHL	CIP
[MAII-3] *	AMP-AMC-LEX-MEM-GEN-KAN-STR	FEP-DOR-CIP-CHL
[NA-1] *	AMP-AMC-LEX-KAN-STR-TET-SXT-CHL	FEP-CTX-DOR-MEM
[NA-2] *	AMP-AMC-CTX-LEX-DOR-IPM-MEM-GEN-KAN-STR-CHL	FEP-CIP
[NA-3] *	AMP-AMC-LEX-DOR-GEN-KAN-STR-CHL	FEP-IPM-MEM-CIP
[NA-4] *	AMP-AMC-LEX-DOR-GEN-KAN-STR-TET-SXT-CHL	FEP
[NA-5] *	AMP-DOR-MEM-GEN-STR-TET-CHL	AMC-FEP
[OA-1] *	AMP-LEX-GEN-SXT-CHL	AMC-FEP-CTX-CFM-MEM-KAN-STR
[OA-2] *	AMP-AMC-LEX-STR-TET-SXT-CHL	KAN
[OA-3] *	AMC-LEX-TET-SXT-CHL	FEP-STR-CIP
[OA-4] *	AMP-AMC-CTX-LEX-TET-SXT-CHL	MEM-GEN-KAN-STR
[PA]	LEX	AMP
[QA-1] *	FEP-LEX-CFM-DOR-MEM-KAN-STR-TET-CHL	AMP-AMC-CTX-GEN
[QA-2]	AMC-CTX-LEX	AMP-FEP-DOR-IPM-MEM-KAN-STR-CIP
[QA-3] *	AMP-AMC-GEN-STR	FEP-KAN-CIP
[QA-4] *	AMP-AMC-LEX-GEN-KAN-STR	FEP-CTX-CFM-CIP-CHL
[RA-1]	Pan-susceptible	AMP-GEN-KAN-STR
[RA-2]	STR	AMP-KAN
[SA] *	AMP-AMC-FEP-CTX-LEX-CFM-DOR-GEN-KAN-STR-TET-SXT-CHL	No intermediate resistance
[UA-1] *	AMP-AMC-LEX-IPM-GEN-STR-TET-SXT-CHL	FEP-DOR-MEMCIP
[UA-2] *	AMP-AMC-LEX-IPM-GEN-STR-TET-SXT	DOR-CIP
[UA-3] *	AMP-AMC-STR-TET-SXT	DOR-MEM-CIP
[VA-1] *	AMP-AMC-CTX-LEX-DOR-MEM-STR-TET-SXT	FEP-IPM-KAN
[VA-2] *	AMP-AMC-LEX-DOR-GEN-KAN-STR-TET-SXT-CHL	CTX-CFM-MEM-CIP
[VA-3] *	AMP-AMC-LEX-GEN-KAN-STR-TET-SXT-CHL	DOR-MEM
[WA-1] *	AMP-AMC-DOR	FEP-IPM-MEM-KAN-STR
[WA-2] *	AMP-AMC-LEX-DOR-MEM	FEP-IPM-KAN-STR-CIP
[WA-3] *	AMP-LEX-DOR	AMC-FEP-CTX-IPM-MEM-GEN-KAN-STR

^1^ The label of the *E. coli* isolates (ID) refers to the associated cheese sample and the isolate’s number. For example, [B-1] indicates the *E. coli* isolate #1 that was retrieved from the Akkawi sample B. ^2^ Resistance to antibiotics was determined using the disk diffusion assay and the Clinical and Laboratory Standards Institute (CLSI) and the European Committee on Antimicrobial Susceptibility Testing (EUCAST) guidelines. AMP, ampicillin; AMC, amoxicillin plus clavulanic acid; FEP, cefepime; CTX, cefotaxime; LEX, cephalexin; CFM, cefixime; DOR, doripenem; IPM, imipenem; MEM, meropenem; GEN, gentamicin; KAN, kanamycin; STR, streptomycin; TET, tetracycline; CIP, ciprofloxacin; NOR, norfloxacin; SXT, trimethoprim-sulfamethoxazole; CHL, chloramphenicol. The antibiotics in the resistance profiles are arranged according to the order of antibiotics/classes listed in the CLSI guidelines. Isolates with (*) are classified as multidrug-resistant, showing resistance to at least three classes of antibiotics.

## Data Availability

All the relevant data have been included in this study.

## Supplementary Materials

The following supporting information can be downloaded at: https://www.mdpi.com/article/10.3390/antibiotics12030610/s1, Table S1: The breakpoints that were used to interpret antibiotic susceptibility of the *E. coli* isolated from the cheese samples.
