# Supplementary material for: Assessment of the Microbiological Acceptability of White Cheese (Akkawi) in Lebanon and the Antimicrobial Resistance Profiles of Associated Escherichia coli
Source: Antibiotics (Basel). 2023 Mar 19;12(3):610. doi: 10.3390/antibiotics12030610 (PMC10044863; doi:10.3390/antibiotics12030610)
Supplement: Supplementary file 1 [file antibiotics-12-00610-s001.zip › antibiotics-2228850-supplementary.pdf]

Supplementary File

Table S1: The breakpoints that were used to interpret antibiotic susceptibility of the *E. coli* isolated from the cheese samples.

	Clinical Laboratory Standards Institute (CLSI) M100, 2017<br>Diameter of the Zone of Inhibition (mm)			European Committee on Antimicrobial Susceptibility Testing (EUCAST) Version 13, 2023<br>Diameter of the Zone of Inhibition (mm)		
Antibiotic agent	Susceptible (S)	Intermediate Resistance (I)	Resistant (R)	Susceptible (S)	Intermediate Resistance (I)	Resistant (R)
Ampicillin (10 µg)	≥ 17	14–16	≤ 13	≥ 14	-	< 14
Amoxicillin-Clavulanic acid (20/10 µg)	≥ 18	14–17	≤ 13	≥ 19	-	< 19
Cefepime (30 µg)	≥ 25	19–24	≤ 18	≥ 27	-	< 24
Cefotaxime (30 µg)	≥ 26	23–25	≤ 22	≥ 17	-	< 17
Cefixime (5 µg)	≥ 19	16–18	≤ 15	≥ 20	-	< 17
Doripenem (10 µg)	≥ 23	20–22	≤ 19	≥ 24	-	< 21
Imipenem (10 µg)	≥ 23	20–22	≤ 19	≥ 22	-	< 19
Meropenem (10 µg)	≥ 23	20–22	≤ 19	≥ 22	-	< 22
Gentamicin (10 µg)	≥ 15	13–14	≤ 12	≥ 17	-	< 17
Kanamycin (30 µg)	≥ 18	14–17	≤ 13	-	-	-
Streptomycin (10 µg)	≥ 15	13–14	≤ 12	-	-	-
Tetracycline (30 µg)	≥ 15	12–14	≤ 11	≥ 18	-	< 18
Ciprofloxacin (5 µg)	≥ 21	16–20	≤ 15	≥ 25	22–24	< 22
Norfloxacin (10 µg)	≥ 17	13–16	≤ 12	≥ 24	-	< 24
Trimethoprim-sulfamethoxazole (1.12/23.75 µg)	≥ 16	11–15	≤ 10	≥ 14	-	< 11
Chloramphenicol (30 µg)	≥ 18	13–17	≤ 12	-	-	-
